# Characteristics of the Foot Static Alignment and the Plantar Pressure Associated with Fifth Metatarsal Stress Fracture History in Male Soccer Players: a Case-Control Study

**DOI:** 10.1186/s40798-017-0095-y

**Published:** 2017-08-07

**Authors:** Sho Matsuda, Toru Fukubayashi, Norikazu Hirose

**Affiliations:** 1 Graduate School of Sport Sciences, Waseda University, 2-579-15 Mikajima, Tokorozawa Saitama, 359-1192 Japan; 2 Faculty of Sport Sciences, Waseda University, Saitama, Japan

## Abstract

**Background:**

There is a large amount of information regarding risk factors for fifth metatarsal stress fractures; however, there are few studies involving large numbers of subjects.

This study aimed to compare the static foot alignment and distribution of foot pressure of athletes with and without a history of fifth metatarsal stress fractures.

**Methods:**

The study participants comprised 335 collegiate male soccer players. Twenty-nine with a history of fifth metatarsal stress fractures were in the fracture group and 306 were in the control group (with subgroups as follows: 30 in the fracture foot group and 28 in the non-fracture group). We measured the foot length, arch height, weight-bearing leg–heel alignment, non-weight-bearing leg–heel alignment, forefoot angle relative to the rearfoot, forefoot angle relative to the horizontal axis, and foot pressure.

**Results:**

The non-weight-bearing leg–heel alignment was significantly smaller and the forefoot angle relative to the rearfoot was significantly greater in the fracture foot group than in the control foot group (*P* = 0.049 and *P* = 0.038, respectively). With regard to plantar pressure, there were no significant differences among the groups.

Midfield players had significantly higher rates of fifth metatarsal stress fracture in their histories, whereas defenders had significantly lower rates (chi-square = 13.2, *P* < 0.05). There were no significant differences in the frequency of fifth metatarsal stress fractures according to the type of foot (kicking foot vs. pivoting foot) or the severity of ankle sprain.

**Conclusions:**

Playing the midfield position and having an everted rearfoot and inverted forefoot alignment were associated with fifth metatarsal stress fractures. This information may be helpful for preventing fifth metatarsal stress fracture recurrence. More detailed load evaluations and a prospective study are needed in the future.

## Key Points

- The results of the present study suggest that an everted rearfoot and inverted forefoot alignment are associated with a history of fifth metatarsal stress fracture.
- Plantar pressure did not differ between the fifth metatarsal stress fracture group and the control group.
- Midfield players had significantly higher rates of fifth metatarsal stress fracture, whereas defenders had significantly lower rates.

## Background

A fifth metatarsal stress fracture (MT-5 fracture) is a common injury in soccer players. In fact, a previous investigation of a European soccer league found that 78% of stress fractures occurring in professional soccer players involved the fifth metatarsal bone. The incidence was 0.037–0.04/1000 exposure hours [1, 2] and 0.10–0.12/1000 athlete exposures in Japan [3].

The MT-5 fracture is well known and requires a long period to recover from [4–14]. Moreover, MT-5 fractures may not achieve union because of poor blood flow around the injured region (the proximal diaphysis) [15]. For instance, surgical treatment requires a shorter period (15.2 ± 10.5 weeks) before regaining the ability to play than conservative treatment (26.3 ± 11.0 weeks) [16]. Therefore, occasional surgical treatment is recommended for athletes [7, 11, 13, 16–18]. However, even if the MT-5 fracture is treated surgically, it takes at least 3–8 months before the individual can play sports again. Therefore, this injury has a negative impact on performance, and prevention of its occurrence is important.

Moreover, MT-5 fractures are well known for their high recurrence rate [14]. The MT-5 fracture recurrence rate is 25%, which is much higher than that of hamstring strains (13%) [19] or ankle sprains (10.3%) [20]. Therefore, in addition to preventing the initial injury, it is important to prevent injury recurrence.

- As van Mechelen et al. proposed, identifying the mechanism and risk factors of the targeted injury is necessary for preventing injury [21]. A previous retrospective study suggested that an inverted rearfoot, which can be examined using radiographs, is a risk factor for MT-5 fracture [10, 22]. However, this screening procedure is difficult to generalize because X-ray assessment requires specific locations, equipment, and skilled technicians in the field. To resolve this issue, easier, alternative protocols for evaluating risk factors of MT-5 fractures, such as high medial longitudinal arch height [23] and plantar pressure [24] have been proposed. However, no consensus regarding risk factors for MT-5 fractures has been established because of a lack of studies with large numbers of subjects.

This study aimed to identify the possible risk factors for recurrence of MT-5 fractures. To clarify this issue, we compared the static foot alignment and distribution of foot pressure during leg calf raise exercises of players with a history of MT-5 fractures with those of healthy players. The calf raise task demonstrates loading on the forefoot. Danahue et al. reported that peak fifth metatarsal strain was 80% through the stance of walking (during forefoot loading). It has been hypothesized that players with a history of MT-5 fractures exhibit rearfoot inversion alignment and that their foot pressure is biased to the lateral region of the foot.

## Methods

All study protocols were approved by the Ethics Committee on Human Research of the university. This study conforms to the Declaration of Helsinki. All subjects were fully informed of the procedures and the purpose of this study and provided written informed consent. The participants were free to withdraw from participation at any time without fear of consequences.

The participants comprised 335 collegiate male soccer players from Kanto University Football Association (a level equivalent to NCAA Division 1 or 2). All participants were able to participate in full training sessions with no pain for at least 1 year. Using the results of a questionnaire, they were divided into two groups: 29 in the fracture group (28 unilateral and 1 bilateral) with a history of MT-5 stress fractures diagnosed by an orthopedic surgeon and 306 in the control group (Table 1). In addition, 29 fracture group players were subdivided into the fracture foot (FF) group (30 feet), non-fracture foot (NF) group (28 feet), and control foot (CF; the mean values of bilateral feet data) group (306 players).

**Table 1:** Study subject characteristics

	*N*	Height (cm)	Age (years)	Weight (kg)
Without fracture history	306	173.5 ± 9.9	20.1 ± 1.1	67.9 ± 6.8
With fracture history	29	172.8 ± 5.8	20.0 ± 1.1	67.5 ± 7.0

Data are presented as the mean ± standard deviation

### Measurements and Procedures

The foot length, arch height, weight-bearing leg–heel alignment (W-LHA: same as resting calcaneal stance position), non-weight-bearing leg–heel alignment (N-LHA), forefoot angle relative to the rearfoot (FA-R), forefoot angle relative to the horizontal axis (FA-H), and foot pressure were measured. All measurements were taken by the same physical therapist and were obtained before the soccer season (February to March). In addition, the participant’s dominant foot, history of ankle sprain, ankle sprain severity, and playing position were obtained using a questionnaire.

#### Arch Ratio

The selected method to measure arch height (the bony arch index) was the anthropometric technique described by Cowan. The foot length (back of the heel to the tip of the toe) and navicular height were measured using a ruler [25]. The arch ratio was defined as the arch height divided by the foot length. The intraclass coefficient (ICC) values were 0.94 for intrarater reliability and 0.89 for interrater reliability.

#### Rearfoot and Forefoot Alignment [26–28]

**Fig. 1:**
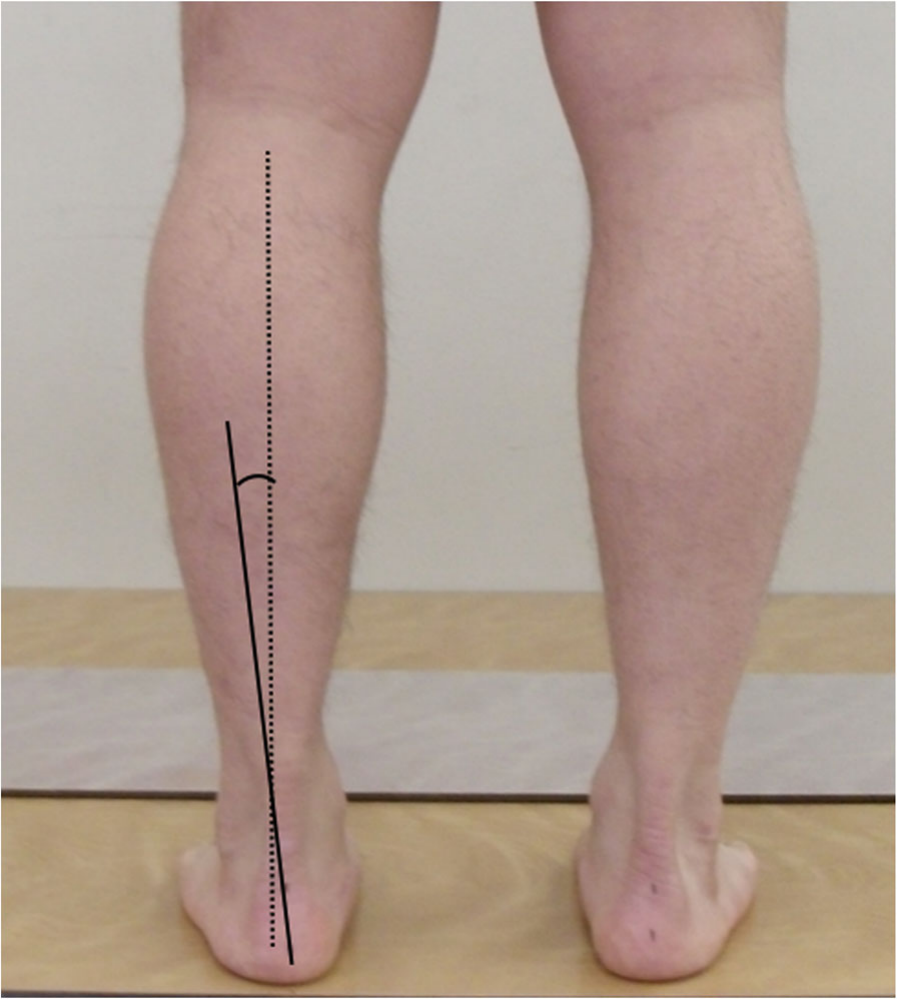
Measurement of W-LHA. Subjects were placed in the weight-bearing position and we measured the angle between the lower leg line and calcaneus line using imageJ

**Fig. 2:**
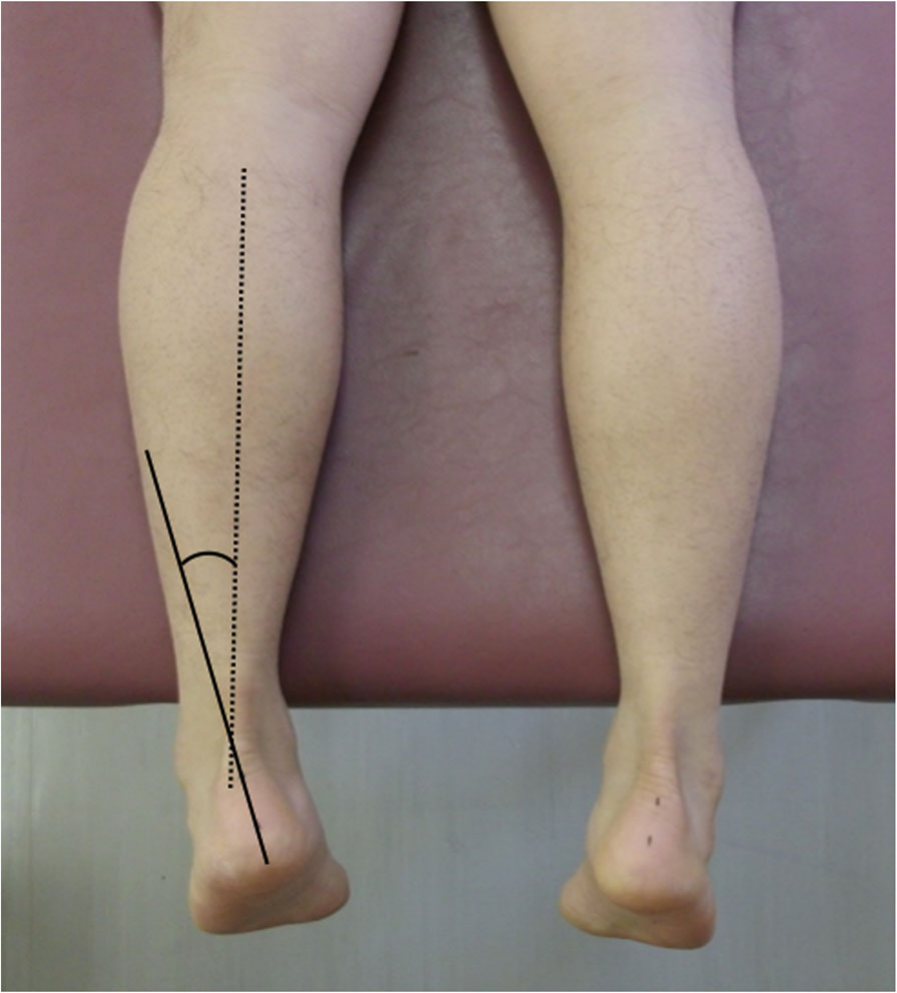
Measurement of N-LHA. Subjects were placed in the non-weight-bearing position and we measured the angle between the lower leg line and calcaneus line using imageJ

Rearfoot and forefoot alignment evaluations were performed as described by Gross et al., and the ICC for intrarater reliability was 0.91 for the forefoot and 0.87 for the rearfoot [26]. We marked two points on the centerline of the calcaneus at the distal and proximal regions and calculated two points on the centerline of the leg and Achilles tendon. Subjects were placed in the weight-bearing position (standing) and non-weight-bearing position (prone) with the hips, knees, and ankles neutral, and photographs were taken from the posterior of the feet (Figs. 1 and 2). A photograph was also taken of all participants’ lower legs and calcanei from the posterior side. Then, we measured the angle between the lower leg line and calcaneus line using image analysis software (ImageJ; US National Institutes of Health, Bethesda, MD, USA).

**Fig. 3:**
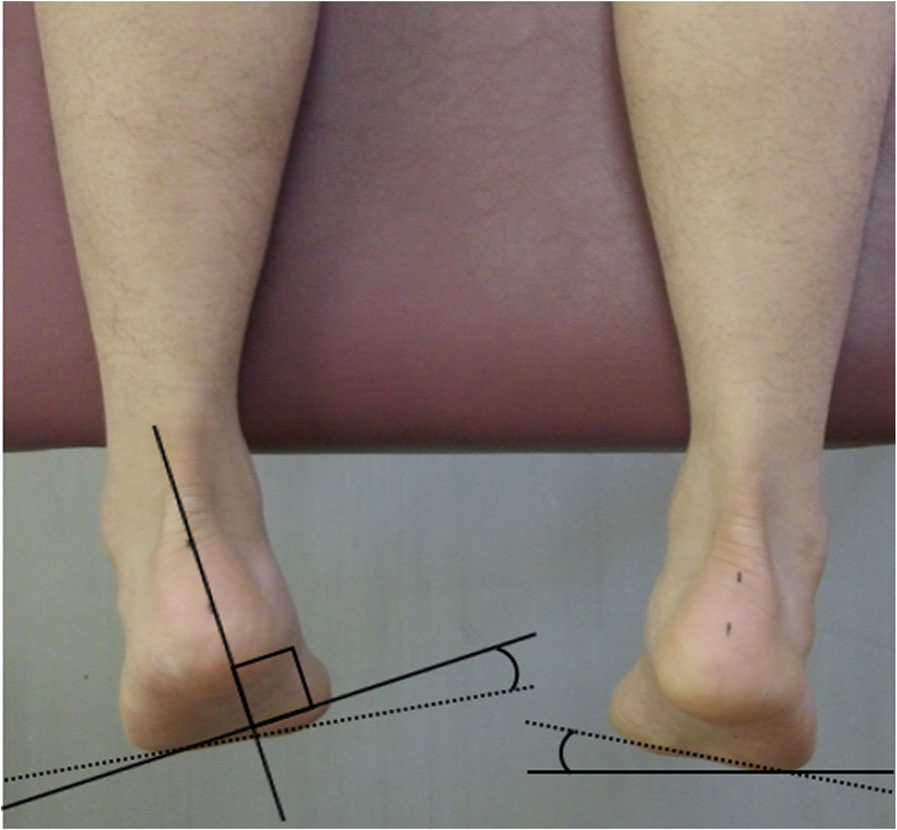
*Left*: measurement of FA-R. *Right*: measurement of FA-H. FA-R: the angle between a line perpendicular to the calcaneal bone axis and a line from the thenar to the hypothenar. FA-H: the angle between the horizontal axis and a line from the thenar to the hypothenar

FA-R is the angle between a line perpendicular to the calcaneal bone axis and a line from the thenar to the hypothenar. FA-H is the angle between the horizontal axis and a line from the thenar to the hypothenar (Fig. 3). For all angles, inversion was indicated with a “+” symbol and eversion was indicated with a “−” symbol.

ICC values for inter-rater reliabilities were 0.97 for N-LHA, 0.90 for W-LHA, 0.99 for FA-H, and 0.93 for FA-R.

#### Plantar Pressure

**Fig. 4:**
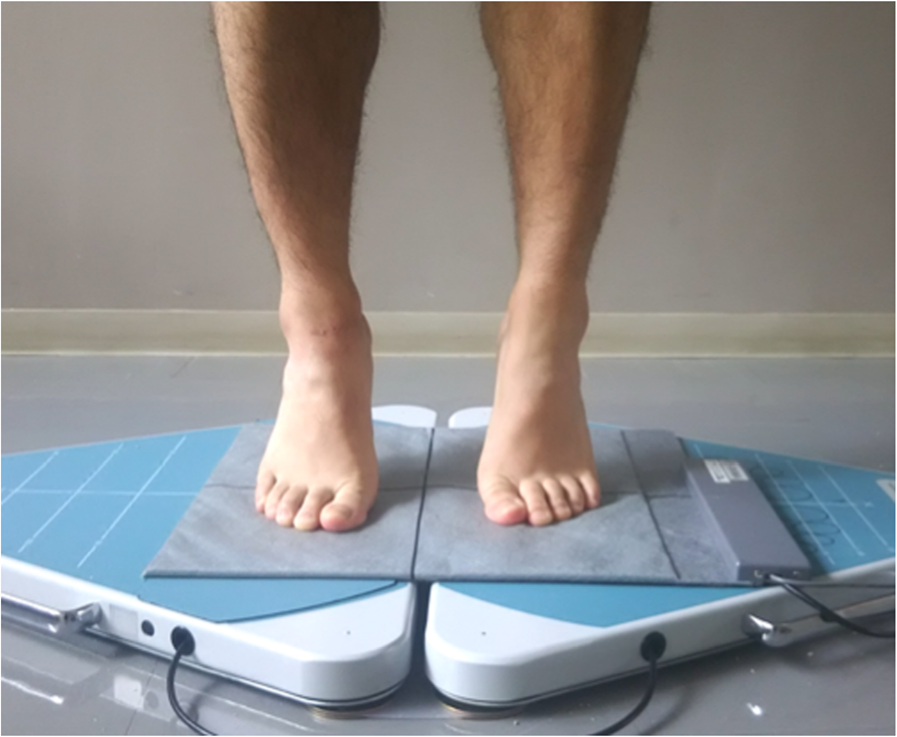
Measurement of the plantar pressure distribution in the heel-raise position. Participants were kept the maximum heel-raise position for 10 s

The center of the plantar pressure was measured using a plate-type Twin Gravicorder GP-6000, and the pressure distribution was measured using a MD-1000 device (Anima Co., Ltd., Tokyo, Japan). While measuring the plantar pressure, participants were kept in two positions: the standing position and the maximum heel-raise position, for 10 s each (Fig. 4). Based on a report that strain on the fifth metatarsal bone increases when loading the forefoot [29], we measured plantar pressure in the heel-raise position. Heel-raise measurements were conducted as follows: we instructed subjects to raise their heel slowly, and if the heel-raise was stable, we started measuring for 10 s. These measurements were taken again for participants who moved during the assessment. The mean value of the center of plantar pressure was determined.

**Fig. 5:**
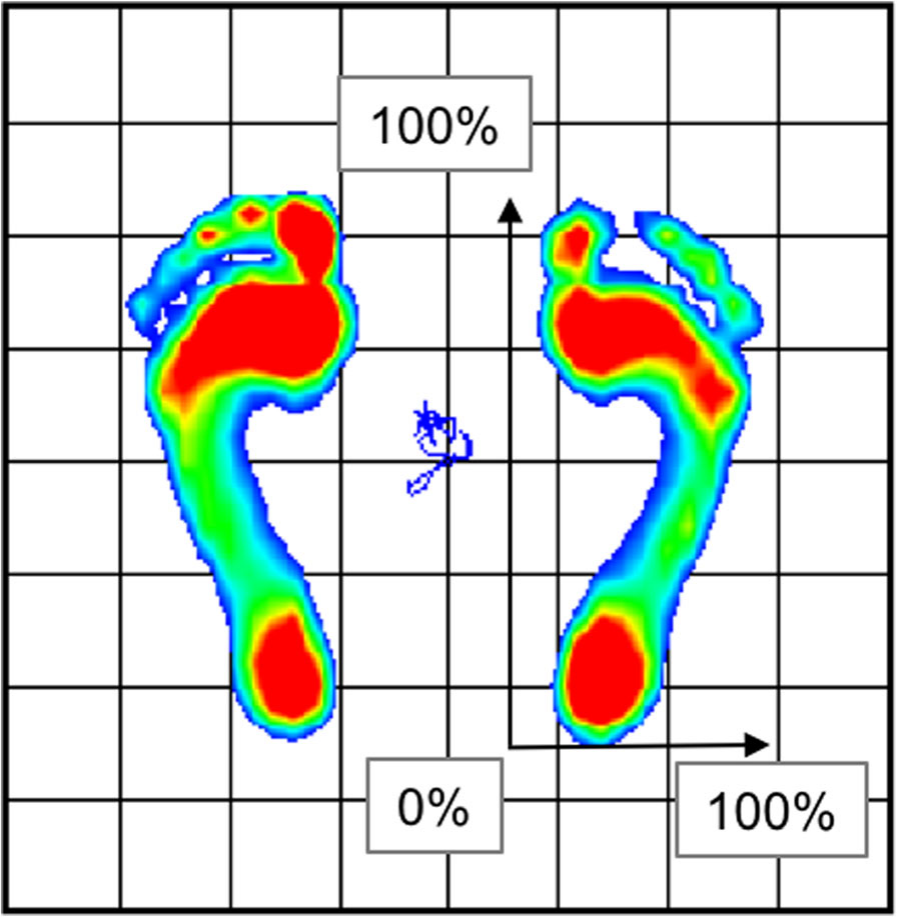
Distribution of plantar pressure. P-A of 0% is the posterior end of the heel and M-L of 0% is the medial end of the foot

The distribution of plantar pressure was defined as from the posterior to anterior (P-A) and medial to lateral (M-L) of the foot, where P-A of 0% is the posterior end of the heel and M-L of 0% is the medial end of the foot (Fig. 5).

ICC values were 0.84 for M-L during standing, 0.73 for P-A during standing, 0.82 for M-L during heel-raise, and 0.69 for P-A during heel-raise.

### Statistical Analysis

History of ankle sprain was compared among the FF, NF, and CF groups using the chi-square test. The chi-square test was also performed for positional differences and measurement of the kicking foot. The Shapiro-Wilk test was used to confirm the normal distribution of data. The foot alignment and plantar pressure in the FF, NF, and CF groups were compared using one-way analysis of variance (ANOVA) and Tukey-Kramer post hoc tests. All statistical analyses were performed using IBM SPSS statistics version 22 (IBM Corp., Armonk, NY, USA). Statistical significance was set at *P* < 0.05.

## Results

**Fig. 6:**
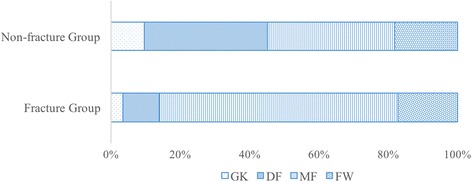
Comparisons of the playing positions. *GK* goal keeper, *DF* defender, *MF* midfielder, *FW* forward. Midfield players had significantly higher rates of MT-5 fractures and defenders had significantly lower rates

The results of the questionnaire indicated that those with a history of fractures comprised one goalkeeper (4%), three defenders (10%), 20 midfielders (69%), and five forward players (17%). The non-fracture group comprised 29 goalkeepers (9%), 109 defenders (36%), 112 midfielders (37%), and 56 forward players (18%). Midfield players had significantly higher rates of MT-5 fractures, and defenders had significantly lower rates (chi-square = 13.2, *P* < 0.05) (Fig. 6).

**Fig. 7:**
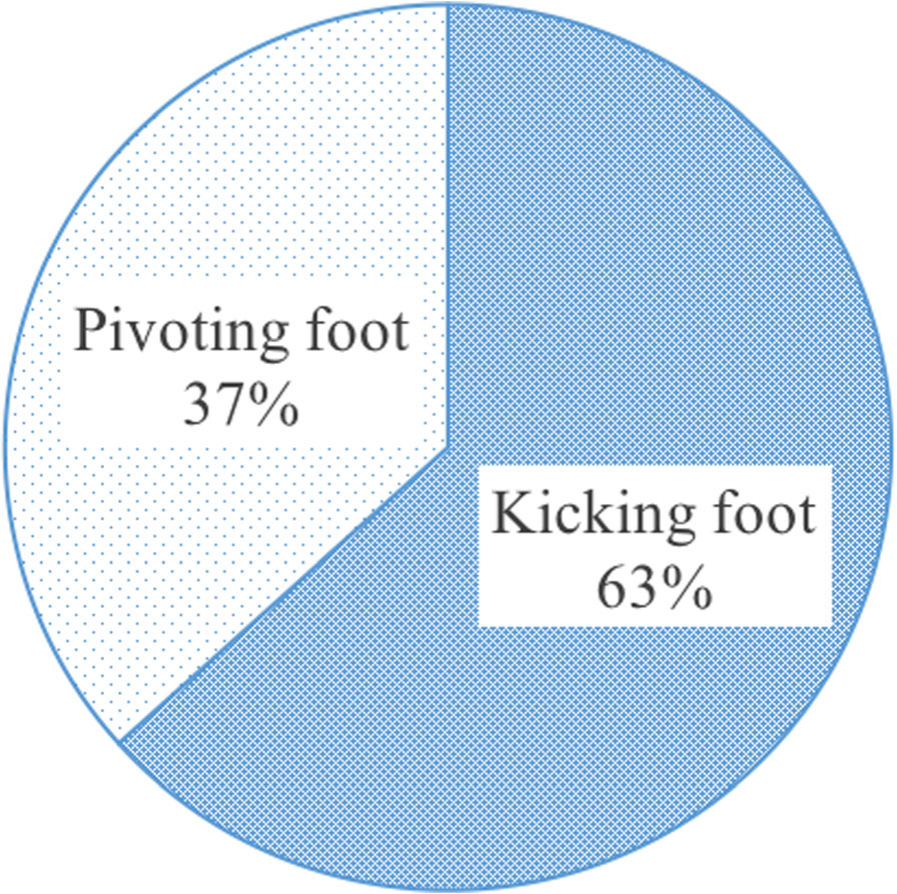
Comparison between the kicking foot and pivoting foot. There were no significant differences

**Fig. 8:**
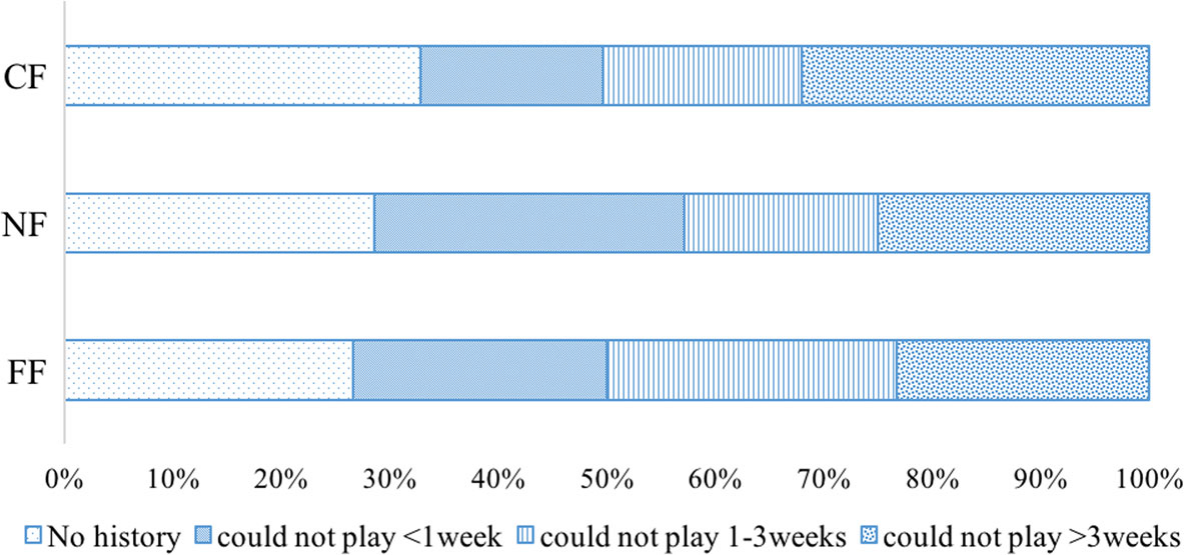
Comparisons according to ankle sprain severity. There were no significant differences

There were no significant differences in the frequency of 5-MT fractures according to the type of foot (kicking foot vs. pivoting foot) or ankle sprain severity (Figs. 7 and 8).

Regarding alignment, N-LHA was significantly smaller and FA-R was significantly larger in the FF group than in the CF group (*P* = 0.049 and *P* = 0.038, respectively). In addition, N-LHA was significantly smaller in the FF group than in the NF group (*P* = 0.042). There were no significant differences in other alignment data among the groups (Table 2).

**Table 2:** Alignment data among the groups

	CF	NF	FF	P (CF-FF)	P (NF-FF)
Arch ratio (%)	17.0 ± 2.3	16.6 ± 2.1	17.3 ± 2.2	0.84	0.53
W-LHA (°)	−4.2 ± 3.1	−2.0 ± 3.5	−3.8 ± 2.2	0.82	0.11
N-LHA (°)	8.5 ± 5.4	9.7 ± 6.0	5.8 ± 5.0	0.049^a^	0.042^a^
FA-R (°)	4.4 ± 6.5	4.5 ± 5.3	7.1 ± 5.8	0.038^a^	0.35
FA-H (°)	15.1 ± 6.6	16.4 ± 8.7	14.1 ± 6.6	0.76	0.43

Data are presented as the mean ± standard deviation

^a^Significant difference

Regarding plantar pressure, there were no significant differences among the groups (Table 3).

**Table 3:** Distribution of the center of pressure (Average for 10 s)

	CF	NF	FF	P (CF-FF)	P (NF-FF)
Standing M-L (%)	48.3 ± 3.5	47.6 ± 3.1	47.9 ± 4.7	0.397	0.253
Standing P-A (%)	44.4 ± 5.8	41.3 ± 8.8	41.0 ± 7.9	0.10	0.98
Heel-raise M-L (%)	46.8 ± 4.8	46.9 ± 7.3	45.3 ± 6.4	0.59	0.09
Heel-raise P-A (%)	75.2 ± 3.1	75.5 ± 3.2	74.8 ± 2.4	0.15	0.39

Data are presented as the mean ± standard deviation

## Discussion

This study aimed to clarify the characteristics of playing position, foot alignment, and plantar pressure during heel raise tasks of players with a history of MT-5 fractures. Our main findings were that the foot with a history of MT-5 fractures (FF) exhibited reduced LHA during non-weight-bearing conditions compared with the healthy foot or the feet of controls; however, there was no difference in LHA under weight-bearing conditions. Additionally, the FF group exhibited greater forefoot inversion relative to the rearfoot than did controls. No difference in foot pressure was identified.

In this study, everted rearfoot and inverted forefoot alignments were only observed during non-weight-bearing conditions. Monaghan et al. reported that alignment in the non-weight-bearing position reflects kinematics during walking and running [27, 28]. Moreover, during cutting and turning movements, the forefoot contacts the ground first; therefore, an inverted forefoot position may cause ground contact with the lateral part of the forefoot. The inverted forefoot may then create a high load at the lateral plantar part of the forefoot. It may be useful to note the alignment in the non-weight-bearing position in future studies. Contrary to expectations, the rearfoot performed everted, rather than inverted. After surgery, many players make contact with the ground on the medial side of their foot while walking because of pain and fear. Moreover, the load on the outside of the foot may have been corrected by rehabilitation. It is possible that several postoperative factors influenced this everted rearfoot alignment.

Under weight-bearing conditions, previous literature has reported that players with a history of MT-5 fractures tended to have inverted rearfoot alignment [10, 22]. In the previous study, radiographs were performed to measure the weight-bearing rearfoot alignment, and lateral radiographs were used to calculate the calcaneal pitch angle. However, in our study, the pictures for evaluation were taken from the posterior side to measure the angle of the rearfoot. This difference in measurement methods may have caused contrary results.

There were also no significant differences in plantar pressure among the groups. Hetsroni et al. researched plantar pressure in athletes who had sustained a proximal fifth metatarsal stress fracture during gait and reported that the loading pressure of the lateral part of the forefoot was low [24]. It is possible that a player who has experienced a fracture cannot bear a load on the lateral part of the forefoot because of fracture pain. In addition, the load on the outside of the foot may have been corrected by rehabilitation through weight-bearing training. In contrast to our findings, Azevedo et al. reported that young soccer players present with asymmetries in plantar pressure in the hallux, fifth metatarsal, and medial rearfoot specifically because of soccer. These contradictory results may have been due to differences between the evaluation procedures. Further studies are needed to analyze in detail the plantar pressure of each region of the foot [30].

There were no significant differences in arch ratio between the FF, CF, and NF groups. Teyhen et al. and Wong et al. reported that the center of pressure was more lateral in the high arch group during gait stance [31, 32]. However, this current study did not demonstrate a relationship between MT-5 fractures and high arch ratio. In future studies, the influence of the arch ratio on plantar pressure during cutting, jumping, and turning tasks should be determined.

In addition, it is necessary to evaluate load movement before MT-5 fractures and one-leg movements such as cutting and turning.

Midfielders had significantly higher rates of MT-5 fractures whereas defenders had significantly lower rates. There are few preliminary research reports on the relationship between playing position and MT-5 fractures. Dellal et al. investigated the physical activity of soccer players and reported that the running distance was significantly greater for midfielders than for defenders [33]. Orendurff et al. reported that the bending moment of the fifth metatarsal increased during the running acceleration phase [34]. Therefore, MT-5 fractures may be associated with increased running distance. In addition, MT-5 fractures are common in soccer players because of the combination of long running distance and cutting and turning movements, which are associated with MT-5 fracture. In future studies, movement characteristics and practice intensity for each position should be investigated.

There was no significant difference in the occurrence of MT-5 fracture between the kicking foot and pivoting foot. Elite players use not only the dominant foot but also the non-dominant foot for kicking; therefore, the proportions of right foot use and left foot use for kicking will need to be investigated.

A relationship between ankle sprain history and MT-5 fracture was not noted. In the present study, only ankle sprain severity was considered. It may be necessary to investigate the relationship between lateral instability of the ankle and MT-5 fracture.

## Conclusion

The results of the present study suggest that playing a midfield position and everted rearfoot and inverted forefoot alignments were associated with 5-MT fractures. These results provide evidence that alignment assessments may be helpful in risk screening for fifth metatarsal stress fractures. A more detailed load evaluation and prospective study are needed in the future.

## Abbreviations

CF: Control foot
FA-H: Forefoot angle relative to the horizontal axis
FA-R: Forefoot angle relative to the rearfoot
FF: Fracture foot
ICC: Intraclass coefficient
LHA: Leg–heel alignment
M-L: Medial to lateral
MT-5 fracture: Fifth metatarsal stress fracture
NCAA: National collegiate athletic association
NF: Non-fracture foot
N-LHA: Non-weight-bearing leg–heel alignment
P-A: Posterior to anterior
W-LHA: Weight-bearing leg–heel alignment
